# Influence of Tempering Temperature on Mechanical and Rotational Bending Fatigue Properties of 40CrNi2MoE Steel

**DOI:** 10.3390/ma17061377

**Published:** 2024-03-17

**Authors:** Chang-Da Yao, Yong Li, Zhi-Wei Zang, Xin-Yang Li, Shun Han

**Affiliations:** 1 Institute of Special Steels, Central Iron and Steel Research Institute Co., Ltd., Beijing 100081, China; ambuacheyaocd@proton.me (C.-D.Y.);; 2 Tianjin Heavy Industries Research & Development Co., Ltd., Tianjin 300457, China

**Keywords:** tempering temperature, modified Crussard–Jaoult method, rotational bending fatigue

## Abstract

In order to examine the mechanical properties and rotational bending fatigue performance of 40CrNi2MoE steel subsequent to tempering at varying temperatures, the steel specimen was subjected to tempering within the range of 400~460 °C. SEM, EBSD, and TEM were used to analyze the microstructure as well as precipitates. The strain hardening law was studied using the modified Crussard–Jaoult method. Investigations were undertaken to reveal the rotational bending fatigue life with respect to the tempering temperature. The findings indicate that the strength and fatigue life of the examined steels exhibit a decline as the tempering temperature increases, with the primary factor affecting this trend being the alteration in dislocation density. No notable impact on the fatigue fracture morphology exerted by tempering temperature was found within the range of the experiment. The C–J model analysis reveals that the work-hardening behavior of the trial steels is influenced by dislocations and the second phase.

## 1. Introduction

40CrNi2MoE steel, belonging to the nickel–chromium–molybdenum series of low-alloy ultra-high-strength steels, epitomizes exemplary material characteristics, including notable strength, toughness, and favorable hardenability. As a result, its usage spans a wide range of applications, prominently in the manufacture of vital components like aircraft landing gears [1,2].

Extensive investigations have been devoted to exploring the influence of various heat treatment protocols, specifically austenitizing temperature, tempering temperature, and cryogenic treatment, on the metallurgical structure and mechanical properties of 40CrNi2MoE steel. Manokaran [3] has studied the influence of different melting routes (vacuum degassing, electro-slag remelting, and vacuum arc remelting) on toughness behavior, which suggests that the homogeneously distributed fragmented carbides in ESR may cause its lesser embrittlement.

It has been demonstrated that there is an enhanced fracture toughness of 40CrNi2MoE steel through augmented residual austenite content while eliminating twinned martensite [4,5,6,7]. Conversely, elevating tempering temperature prompts the decomposition of lath martensite and the coarsening of carbides, thereby detrimentally influencing the strength of the material [8,9,10]. With the increase in tempering temperature, the type of precipitated carbides in steel will be transformed. Low-temperature tempering below 300 °C produces transition carbides η, and when tempered above 325 °C, η carbides are gradually transformed into cementite and the elemental redistribution of cementite occurs. However, when the tempering temperature is increased to above 575 °C, the coarsened cementite reduces the fracture properties of the steel [11].

Various strength–toughness 40CrNi2MoE steels obtained by adjusting tempering temperature have different crack propagations, which is determined by the competitive relationship between stress and strain around the crack tip. As toughness increases, the crack extension mechanism changes from stress-controlled to strain-controlled, and the fracture morphology transitions from a cleavage plane to dimples [12].

To preserve the steel’s strength while simultaneously bolstering its toughness, the literature posits the adoption of an isothermal quenching process to achieve a dual-phase microstructure comprising bainite and martensite, which has more enhanced mechanical performance [13,14,15]. The inclusion of bainite exhibits crack arrest capabilities, while the presence of bainite at the crack front induces stress relaxation effects, culminating in a substantial enhancement in fracture toughness. By applying these refined modifications, a thorough examination of 40CrNi2MoE steel affords a comprehensive analysis of the impact that varying tempering temperatures exert on microstructural attributes, mechanical properties, and the ensuing rotating bending fatigue performance. In addition, toughness can be improved by short-time tempering as well, which may be connected to the reduction in retained austenite decomposition to inter-lath cementite [16].

The constituents employed in aerospace shafts may be exposed to cyclic bending fatigue loads during operational deployment, potentially culminating in fatigue failure. Investigations [17,18,19] have unveiled that, under conditions of elevated cyclic bending loads, fatigue failures primarily emanate from non-metallic inclusions or procedural defects intrinsic to the steel matrix.

This research aims to establish a theoretical framework supported by empirical evidence, thereby facilitating further advancements in augmenting the rotating bending fatigue life of 40CrNi2MoE steel, ultimately contributing to the progression of steel science and engineering.

## 2. Materials and Methods

The present study utilized 40CrNi2MoE steel as its experimental material, which underwent a fabrication process involving vacuum induction melting coupled with vacuum consumable electrode remelting. The detailed chemical composition of the material is presented in Table 1.

**Figure 1:**
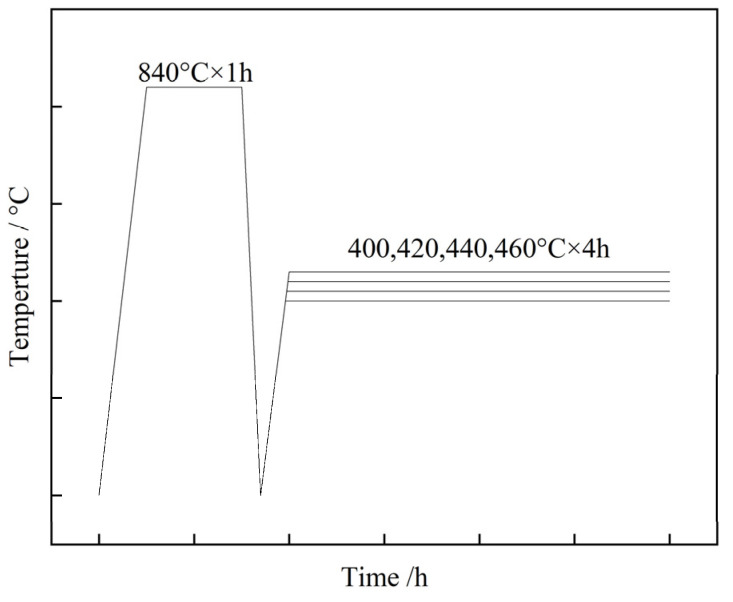
Heat treatment process schematic diagram.

The tested steel was subsequently made into initial blanks, which were then subjected to an oil-quenching procedure at 840 °C for 1 h. Following this, a tempering process ensued, lasting 4 h at 400 °C, 420 °C, 440 °C, or 460 °C. The schematic diagram of the heat treatment process is shown in Figure 1.

To comprehensively evaluate the material’s characteristics, a battery of assessments was undertaken, encompassing both mechanical properties and microstructural attributes. Tensile and impact tests that were undertaken adhered to GB/T 228.1-2021 [20] and GB/T 229-2020 [21]. The microstructure of trial steels was observed by the high-resolution Thermo Fisher Scientific Quanta FEG 650 scanning electron microscope (SEM) and Thermo Scientific Apreo 2C equipped with an EDAX Velocity Super EBSD detector (Waltham, MA, USA). Furthermore, nano-precipitates were scrutinized through a transmission electron microscopy (TEM) analysis. This investigation utilized the FEI Tecnal G2 F20 (Hillsboro, OR, USA) field emission transmission electron microscope, incorporating an energy-dispersive spectroscopy system (EDS) to study the nano-precipitation and dislocations in the trial steels. Dislocation density and austenite content were meticulously ascertained through the X-ray diffraction (XRD) method. Furthermore, the initial blanks underwent precision machining, yielding rotating bending fatigue specimens tailored to accommodate tests.

The rotating bending fatigue test adhered to the standards outlined in GB/T 4337-2015 [22] and was conducted on the QBWP-6000 machine (QianBang, Changchun, China), operating at ambient temperature. The test employed a four-point force methodology with a frequency of 80 Hz, a stress concentration factor (Kt) of 1, and a stress ratio (R) of −1, and the loading waveform adopted was a sinusoidal wave. Upon the completion of the fatigue tests, the fracture surfaces underwent immersion in acetone to undergo thorough ultrasonic cleaning. Subsequently, the fracture morphology was meticulously observed and analyzed utilizing the Quanta FEG 650 SEM (Hillsboro, OR, USA), which was equipped with an X-ray energy dispersive spectrometer.

## 3. Results and Discussion

### 3.1. Microstructure

**Figure 2:**
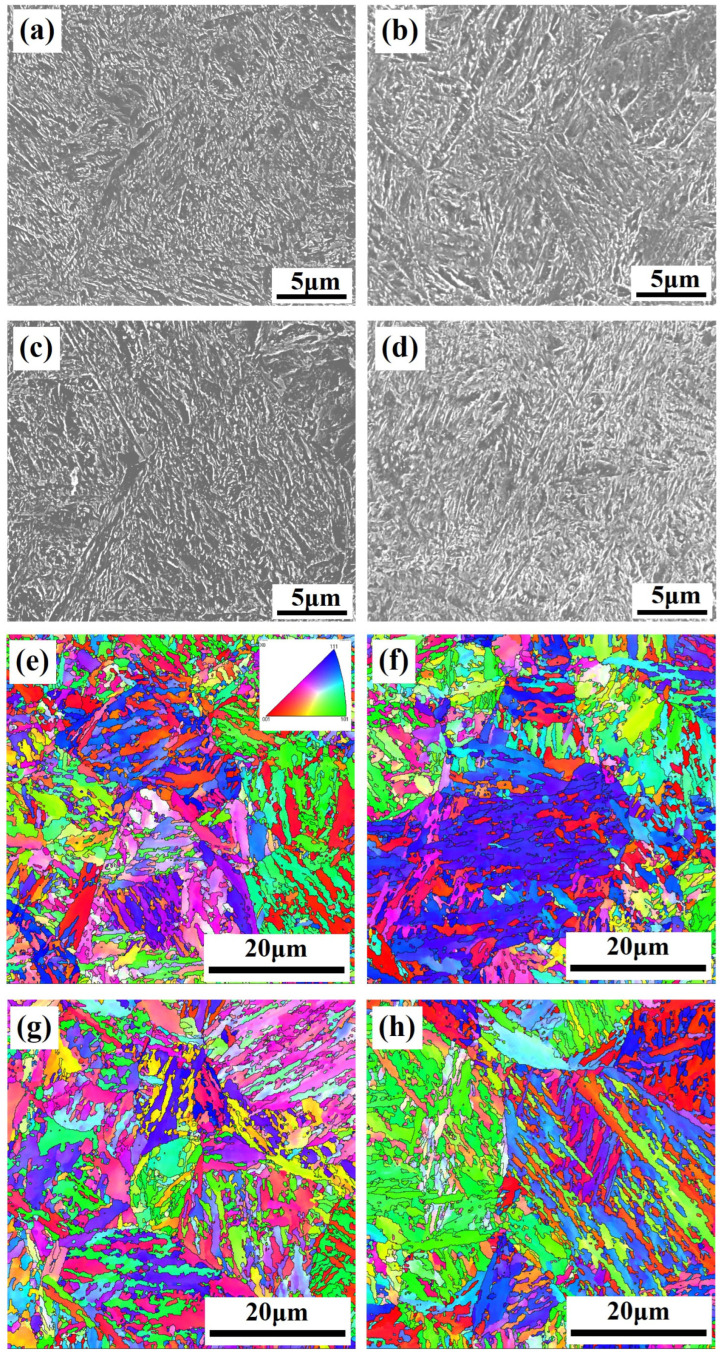
SEM (**a**–**d**) and EBSD (**e**–**h**) of 40CrNi2MoE steel after tempering at various temperatures.

The microstructural characteristics of 40CrNi2MoE steel subsequent to tempering at discrete temperatures of 400, 420, 440, and 460 °C are elucidated by Figure 2. Evidently discernible in the depiction is the dominance of a microstructure comprised of tempered martensite, characterized by lath martensite and precipitated carbides. Analytical findings from XRD underscore austenite content within the specimens post-tempering at varying temperatures, all falling within the narrow spectrum of 0.30% to 0.36%. This result implies that the tempering temperature exerts a modest influence on the austenite content.

**Figure 3:**
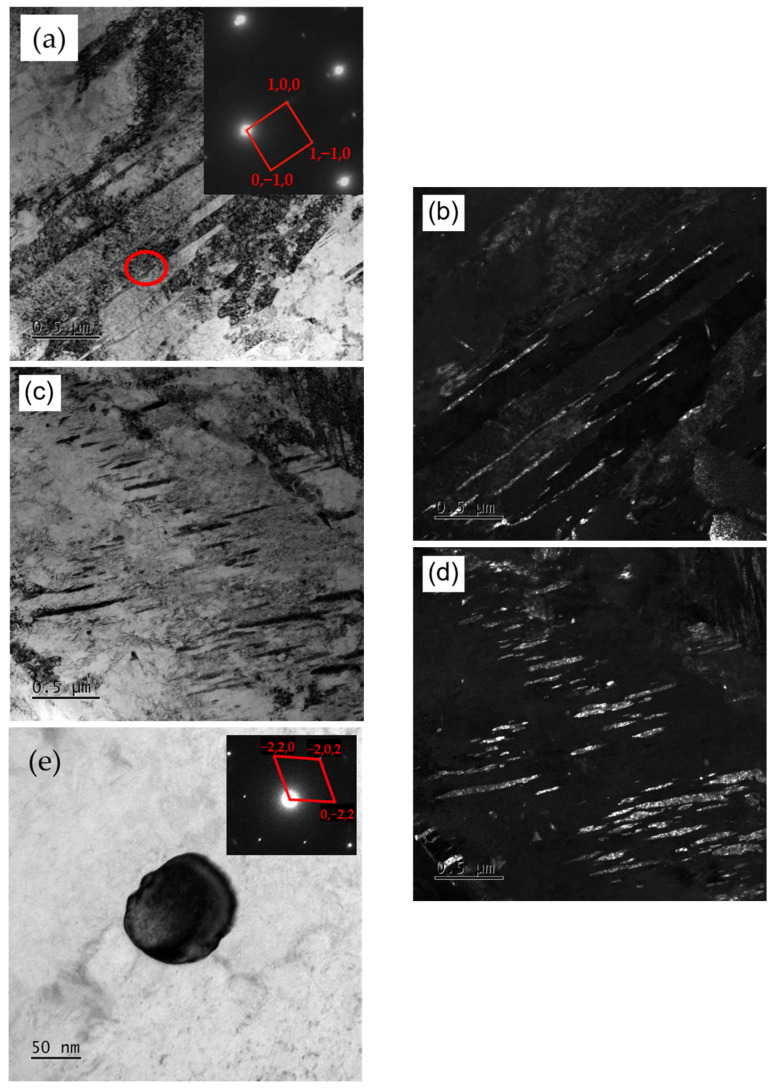
TEM of two carbides in 40CrNi2MoE steel after tempering at 440 °C: (**a**,**b**) BF and DF image of cementite at lath boundaries; (**c**,**d**) BF and DF image of cementite inside martensite lath; (**e**) BF and SAED of M_23_C_6_.

To investigate the nanostructure of the trial steels, TEM investigation was acquired, as illustrated in Figure 3. The TEM bright-field (BF) and dark-field (DF) image reveals the presence of abundant needle-like precipitates intricately embedded along both the boundaries and the interior of the martensitic lath. Simultaneously, a nearly spherical secondary phase within these laths comes into view. Through selected area electron diffraction (SAED), these needle-like precipitates are discerned to be cementite (Fe_3_C). Concurrently, the nearly spherical secondary phase, conspicuous for its Cr enrichment, is found to correspond to M_23_C_6_ carbides. A discernible metamorphosis transpires as the tempering temperature ascends, wherein the needle-like cementite morphology gradually transmutes into rod-like configurations.

### 3.2. Mechanical Properties

**Figure 4:**
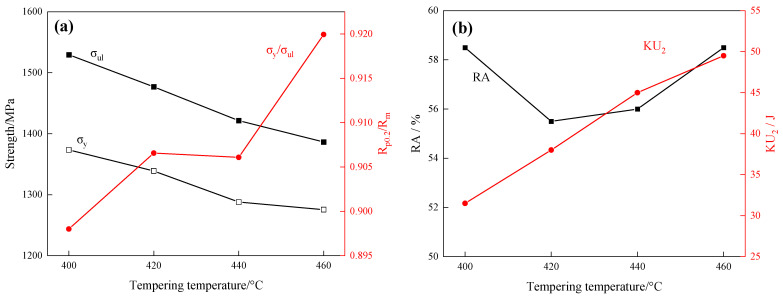
The effect of tempering temperature on mechanical properties of 40CrNi2MoE steel. (**a**) Trends of ultimate strength (σ_ul_), yield strength (σ_y_), and yield ratio (σ_y_/σ_ul_); (**b**) Trends of reduction in area (RA) and impact absorbed energy (KU_2_).

Tensile and impact mechanical property tests were conducted on 40CrNi2MoE steel subjected to various tempering temperatures. The variation in the mechanical properties of the tested steel concerning the tempering temperature is illustrated in Figure 4. Within the temperature spectrum, both ultimate strength (σ_ul_) and yield strength (σ_y_) of the tested steel ebb concomitant to the ascendant tempering temperature. Discernibly, the ultimate strength of the tested steel consistently transcends the 1400 MPa threshold, with the yield ratio (σ_y_/σ_ul_) concurring within the 0.90–0.92 ambit, thus manifesting comprehensive mechanical properties. An observation is the affirmative correlation of tempering temperature with both the resilience against impact absorbed energy (KU_2_) and the yield ratio. And the reduction in area (RA) is slightly influenced by tempering temperature. Upon conjoining the mechanical performance curve with the minutiae gleaned from microscopic scrutiny of the microstructure, an observation ensues: following tempering at 400 °C, the microstructure shows a lath configuration of relative finesse, a concurrence that corresponds with the acme in tensile and yield strengths.

The deformation characteristics of 40CrNi2MoE steel under uniaxial tension at different tempering temperatures were investigated. The Crussard–Jaoult (C–J) analysis [23], renowned for its sensitivity in capturing variations in strain hardening mechanisms at low strains, proved to be a valuable tool for assessing the influence of processing and microstructural factors on the deformation behavior of steels. By employing the modified C–J analytical method to fit the true stress–strain curve, it was possible to discern the strain-hardening properties exhibited by 40CrNi2MoE steel at different stages of development. The modified C–J analytical method is an outgrowth of the Swift equation [24,25,26], which is expressed as $\varepsilon =\varepsilon_{0}+k\cdot \sigma^{n}$. A transformation is achieved by logarithmically processing both facets of this equation, thereby engendering Equation (1):

(1) $$\ln\left(\frac{d\sigma }{d\varepsilon }\right)=\left(1-n\right)\ln\sigma +\ln\left(\mathrm{kn}\right)$$

In Equation (1), *k* is a material constant, while *n* denotes the exponent characterizing strain hardening. The construction of the $\ln\left(\frac{d\sigma }{d\varepsilon }\right)-ln\sigma$ curve is synonymous with the generation of the modified Crussard–Jaoult (C–J) analysis plot [27,28]. The real strains at distinctive junctures within this plot are denominated as transition strains, *ε_t_*.

**Figure 5:**
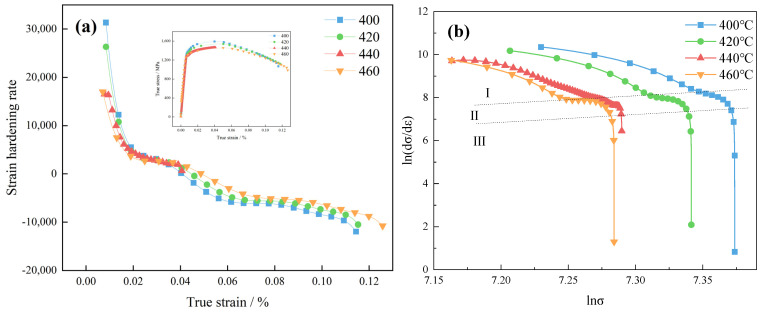
(**a**) Strain hardening rates vs. true strain curves; (**b**) ln(dσ/dε) vs. lnσ of tested steel after tempering at different temperatures.

Figure 5a delineates the trend in the strain-hardening exponent, as inferred from the derivative of the true stress–strain curve post-yield point, fitted with a polynomial function. This tendency unfurls in two discernible phases: an initial precipitous descent succeeded by a gradual abatement until the specimen reaches a state of instability. In proximity to a strain of 0.03, an almost linear plateau manifests, signifying a relative constancy in the strain-hardening exponent at this juncture. Preceding the plateau zone, with escalating tempering temperatures, there is a gradual diminution in the strain-hardening exponent. Conversely, under elevated strains, specimens subject to tempering at heightened temperatures evince an augmented strain-hardening exponent.

In accordance with dislocation theory, when subjected to lower levels of strain, dislocation motion predominantly manifests through the slip mechanism. As the tempering temperature escalates, there is a heightened prevalence of interactions and the subsequent redistribution of high-density dislocation structures. Furthermore, the pinning effect exerted by precipitate particles upon dislocations undergoes augmentation, thereby bestowing increased stability upon dislocation entities and, concurrently, engendering a noteworthy reduction in the population density of mobile dislocations [29].

Consequently, during the initial phase of the strain-hardening curve, there is a conspicuous decrement in the strain-hardening exponent concomitant with the progressive elevation in tempering temperature. This trend aligns harmoniously with the corresponding alterations in dislocation density as ascertained via X-ray diffraction (XRD). Nonetheless, once dislocations commence their motion, the interactions between dislocations and precipitate particles experience attenuation, resulting in a partial augmentation in the population density of mobile dislocations. This, in turn, serves to decelerate the otherwise declining tendency of the strain-hardening exponent.

Furthermore, as a consequence of the spherization process observed in precipitate particles subsequent to tempering at higher temperatures, the interplay with dislocations experiences a further diminishment. The spherization and the interplay will be discussed in Section 3.6. Consequently, following tempering at 460 °C, the amplitude of the upsurge in the mobile dislocation density surpasses that which follows tempering at 400 °C. This phenomenon contributes substantially to the mitigation of the reduction in the strain-hardening exponent. As a result, there is a discernible augmentation in the strain-hardening exponent subsequent to the plateau zone, coinciding with the progressive elevation in the tempering temperature.

Figure 5b elucidates the post-yield evolution of the modified C–J model curve. This curve unfolds across three distinct phases: an initial gradual decline, a precipitous descent, and an intermediate transitional phase. The values of n characterizing each phase, alongside their respective turning point strain $\varepsilon_{t}$, find their summation in Table 2 and Table 3. The n parameter’s magnitude mirrors the robustness of the strain-hardening phenomenon. In the inaugural phase, the efficacy of strain hardening experiences attenuation concomitant with the escalation in tempering temperature. Furthermore, with increasing strain levels, the strain-hardening capacity of specimens subjected to varying tempering temperatures exhibits diminution. The elevation in tempering temperature induces a concurrent decline in the mobile dislocation density inherent to these specimens. Consequently, the work-hardening rate during deformation registers a gradual deceleration, harmonizing with the constriction of the strain range characterizing the initial half of the adapted C–J model curve.

### 3.3. Fatigue Life

**Figure 6:**
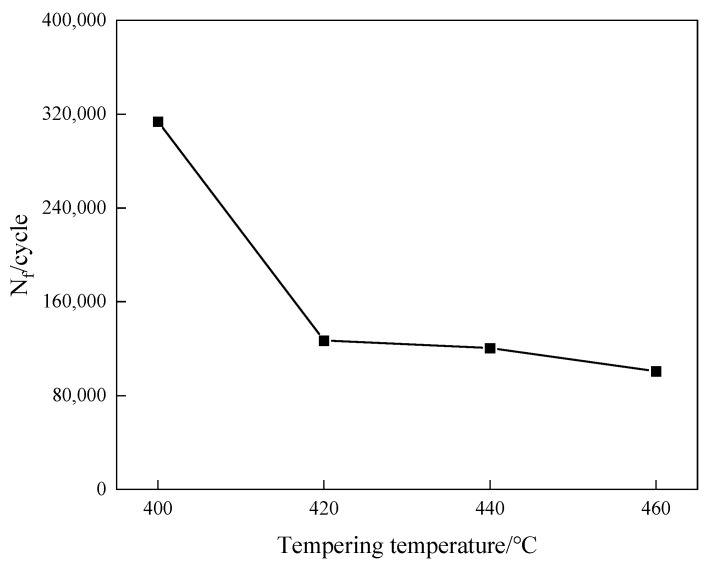
N_f_ vs. tempering temperature.

To investigate the influence of diverse tempering temperatures on the rotating bending fatigue life of 40CrNi2MoE steel, rotational fatigue examinations were executed on smooth experimental specimens subjected to tempering procedures at varying temperatures. The test parameters have been given at the front, and the maximum stress is 800 MPa. The outcomes are delineated in Figure 6, which illustrates the fatigue life’s dependence on tempering temperature in the examination of the trial steels. Notably, within the experimental temperature spectrum, specimens undergoing tempering at 400 °C manifested the most elevated fatigue life, attaining 313,763 cycles. Additionally, with escalating tempering temperatures, a conspicuous decrement in fatigue life ensues, comporting harmoniously with the concurrent trend in strength variation. This concordance illustrates that the factors governing fatigue life are consonant with the determinants of strength.

### 3.4. Fatigue Limit and S-N Curve

**Figure 7:**
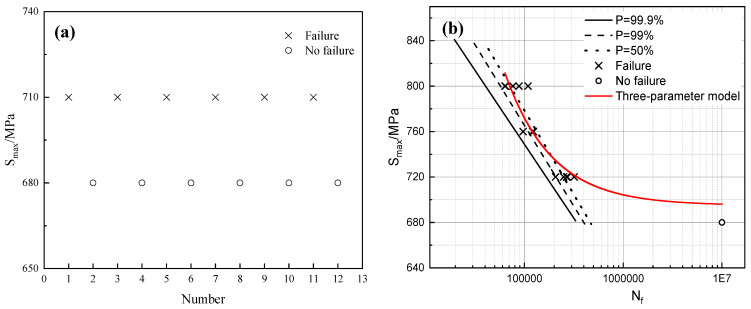
(**a**) Staircase plot; (**b**) P-S-N curves of 40CrNi2MoE steel and three-parameter model fitting curve.

In pursuit of ensuring the steel’s impact toughness without compromising its high strength, a tempering temperature of 440 °C was selected for ascertaining the conditional fatigue limit and constructing the S-N curve. The appraisal of the conditional fatigue limit for 40CrNi2MoE steel, subjected to rotational bending, was executed employing the staircase method, and the resultant staircase plot is showcased in Figure 7a. The initial stress was set at 710 MPa, accompanied by a stress gradient of 30 MPa. A cohort of 12 specimens was enlisted for this endeavor, and the targeted cycle count was set at 10^7^. Computed in accordance with Equation (2), the fatigue limit for 40CrNi2MoE steel is at a commendable 695 MPa. Considering that the yield strength after tempering at 440 °C is 1288 MPa, the ratio of fatigue strength to yield strength is 0.54.

(2) $$\sigma_{\mathrm{RN}}=\frac{1}{m}\sum_{i=1}^{n}V_{i}\sigma_{i}$$

In Equation (2), *m* signifies the number of qualified specimens and *n* conveys the enumeration of stress-level tiers, while *V_i_* and *σ_i_* delineate, in a distinct manner, the quantification of samples and the magnitudes of stress, correspondingly, for the *i*-th level of stress.

The S-N curve of 40CrNi2MoE steel under rotational bending fatigue assessment was determined through a grouped methodology. Three distinct stress levels were selected and five specimens for fatigue evaluation at each tier were subjects. Within the domain of fatigue design and performance assessment, three usual S-N curve models [30,31] are conventionally employed, namely, the exponential function model, the power function model, and the three-parameter model. Notably, comprehensive empirical findings unequivocally support the precision and predictive efficacy of the three-parameter model [32,33]. In the context of the S-N curve, which takes into consideration the fatigue strength denoted as S_i_, the equation for accommodating the three-parameter model can be expressed as Equation (3) [18]:

(3) S=Si1+CNb

Within this equation, *b* denotes the negative gradient of the S-N curve, while C signifies a material constant. As *S* converges toward the threshold S_i_, N asymptotically tends toward infinity. The data points were fitted to the three-parameter model using Origin 2021 software, as depicted by the red curve in Figure 7b, culminating in the formulation of the equation as follows:

(4) S=6951+4217.12N0.916, R2=0.854

It can be seen that the model is fitted better and can reflect the relationship between the fatigue life of 40CrNi2MoE steel and the amount of stress it is subjected to under the test conditions.

Owing to the inherent dispersion in fatigue test data, a one-to-one correspondence between fatigue stress and life is regrettably absent. In actuality, the S-N curve embodies the fatigue life profile for a survival probability of P = 50%. To articulate fatigue life profiles for varying survival probabilities, the formulation of P-S-N curves becomes indispensable. Calculations were meticulously conducted for standard normal deviations and probabilistic fatigue life at survival probabilities of 50%, 99%, and 99.9%, subsequently followed by a linear regression analysis. The resultant P-S-N curve is thoughtfully presented in Figure 7b. From the P-S-N curves, it can be noticed that the fitted line with P = 99.9% is slightly conservative, and all of its predicted fatigue life is shorter than the measured values.

In a scholarly pursuit, Zhang et al. [33] undertook an investigation of SAE 4340 steel subjected to tempering at varying temperatures, yielding an array of mechanical strengths. Their inquiry encompassed the assimilation of fatigue data gleaned from an assortment of high-strength steel alloys, as well as aluminum and copper alloys. Within this ambit, they systematically elucidated the intricate nexus between fatigue strength and tensile strength. The findings unveiled a quadratic correlation between these two pivotal parameters, from which sprang forth a comprehensive fatigue strength formula of import:

(5) $$\sigma_{w}=\sigma_{b}\left(C-P\cdot \sigma_{b}\right)$$

Within the confines of this formula, fatigue strength is symbolized and denoted as $\sigma_{w}$, while $\sigma_{b}$ represents tensile strength, and C and P are both constants. The enduring fatigue characteristics of 40CrNi2MoE steel, gleaned from this empirical endeavor, were harnessed for the fitting of a nuanced three-parameter model curve, thereby yielding insights into the fatigue performance at divergent tempering temperatures. The application of the aforementioned equation, Equation (5), to this fitting procedure bestowed upon them the ensuing mathematical representation:

(6) $$\sigma_{w}=\sigma_{b}\left(1.349-5.709\times 10^{-4}\cdot \sigma_{b}\right)$$

Notably, this model demonstrated a commendable goodness of fit, quantified by a coefficient of R^2^ = 0.892.

### 3.5. Fracture Surface

**Figure 8:**
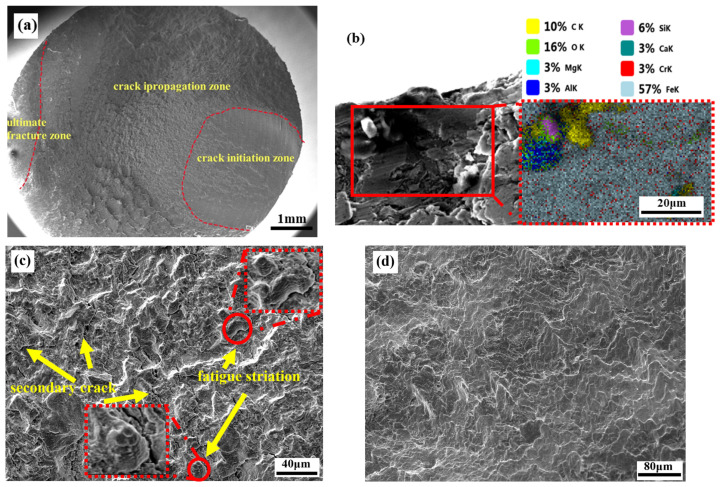
SEM morphology of rotational bending fatigue fracture. (**a**) Macrostructure morphology; (**b**) complex inclusion origin and its component; (**c**) crack propagation region; (**d**) final rupture region.

The fatigue fracture surfaces resulting from rotational bending fatigue present a wealth of valuable information regarding the fracture process. Characteristically, these fatigue fracture surfaces manifest three discernible morphological domains: the fatigue crack origin region, the crack propagation region, and the fatigue final rupture region. Conventionally, the final rupture region is positioned diametrically opposite the origin region. However, due to the accelerated advancement in fatigue cracks in the reverse rotational direction, the location of the rupture region deviates at a certain angular offset in the reverse rotation direction, as elucidated by previous research [34]. The intermediate plane strain region exhibits a radial pattern, as illustrated in Figure 8a. Upon an examination of the fatigue fracture surfaces exhibited by 40CrNi2MoE steel subjected to rotational bending fatigue, a notable revelation emerged: all fatigue cracks originated exclusively from the specimen’s surface. This phenomenon can be ascribed to the persistent oscillation of tensile and compressive stresses, which act upon multiple points of the specimen’s surface in a sinusoidal fashion, thereby culminating in the highest stress amplitude at the surface. Furthermore, the primary origins of crack initiation are traced back to the presence of inclusions, specifically, slag inclusions, and processing defects. These inclusions encompass a blend of Al_2_O_3_, CaO, and MgO, in composite arrangements of two or more constituents, as distinctly delineated in Figure 8b. It is noteworthy that no cracks were initiated solely as a consequence of one kind of inclusion during the fatigue tests. The level of control of inclusions in the steelmaking process needs to be improved.

Within the propagation region, as delineated in Figure 8c, the emergence of a profusion of secondary cracks is discernible. The genesis of these secondary cracks serves the purpose of mitigating stress concentration at the major crack, thus decelerating the advancement velocity of the major crack. In the context of rotational-bending-fatigue-induced fracture surfaces of 40CrNi2MoE steel, the presence of extensive fatigue striations does not manifest within the propagation region. Rather, such fatigue striations, with lengths spanning from 10 to 20 μm, are restricted to isolated diminutive planes. These striations signify the distance traversed by fatigue cracks during each sinusoidal loading cycle, rendering them instrumental for appraising variations in fracture propagation rates through an evaluation of the inter-striation spacing. Figure 8d serves to elucidate the morphological attributes of the final rupture region. Upon scrutiny of the fatigue surfaces, it becomes patently apparent that there exist no noteworthy disparities in the morphology of fractures or the sources of crack initiation across varying tempering temperature conditions. The examination of the fatigue fracture surfaces elucidates that the chosen tempering temperature range in the experimental paradigm is comparatively narrow, thereby falling short in eliciting pronounced distinctions in the morphological attributes of fractures and the origins of initiation.

### 3.6. Dislocation Density

For this section, an investigation was performed on 40CrNi2MoE steel using XRD to measure the alteration in dislocation density with varying tempering temperatures. This study also utilized TEM to examine the presence of dislocations within the martensite laths. The samples were treated by quenching, and tempering for different times.

**Figure 9:**
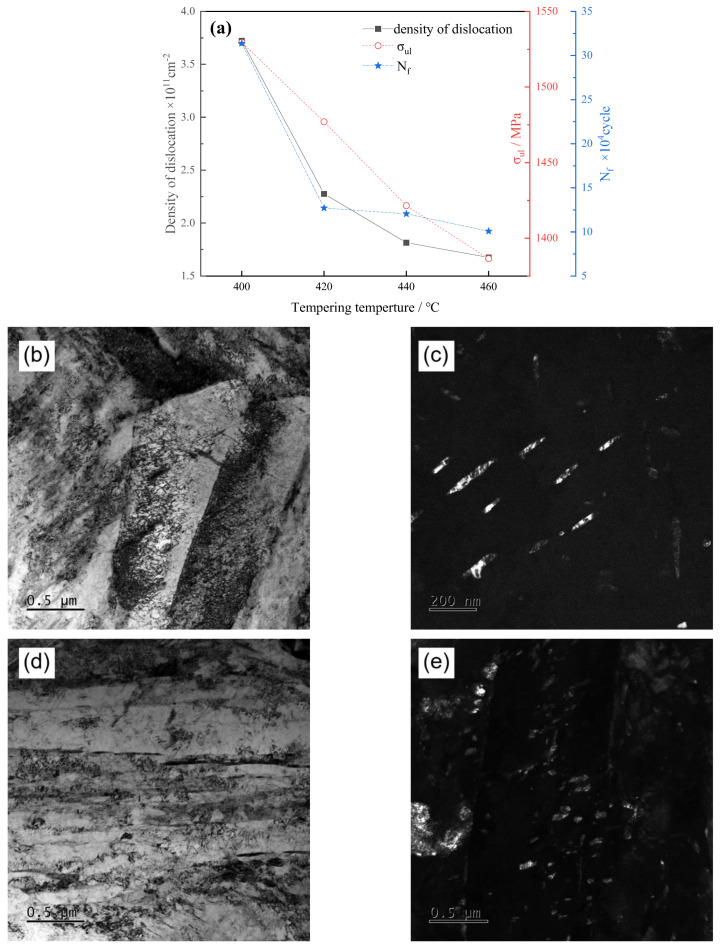
(**a**) Density of dislocation vs. tempering temperature; (**b**,**c**) BF and DF of dislocations and cementite inside lath after tempering at 400 °C, respectively; (**d**,**e**) BF and DF of dislocations and cementite inside lath after tempering at 460 °C, respectively.

The XRD result is shown as depicted in Figure 9a. As tempering temperature rose, there was a gradual reduction in dislocation density within the 40CrNi2MoE steel. This phenomenon can be attributed to the release of residual stresses during the tempering process. By alleviating the resistance to dislocation movement, this stress release facilitated increased dislocation annihilation and subsequently caused a decline in dislocation density [35]. Concurrently, as the tempering temperature rises, a marginal portion of the martensitic phase undergoes an inverse transformation, giving rise to the formation of reverse-transformed austenite. The higher cubic symmetry and atomic packing density of the austenite lattice structure made it more susceptible to dislocation slip, resulting in a further decrease in dislocation density.

Furthermore, it can be seen that the increase in tempering temperature reduces the entangled dislocations within the martensitic slats of the test steel in the TEM BF images (Figure 9b,d). TEM images revealed that a transformation occurred in the precipitated carbides within the laths as the tempering temperature increased. This transformation changed their elongated needle-like shapes into shorter rod-like forms, leading to reduced hindrance to dislocation motion [36]. Consequently, this transformation promoted dislocation annihilation and caused a decrease in dislocation density.

Upon comparing the effects of tempering temperature on dislocation density, tensile strength, and fatigue life, it was observed that the sample tempered at 400 °C exhibited the highest dislocation density and the highest strength, as well as the longest fatigue life. Nevertheless, as the tempering temperature continued to rise, a concurrent decrease was observed in both dislocation density and the parameters of strength and fatigue life. Consequently, it can be inferred that the deterioration of tensile strength and fatigue life resulting from elevated tempering temperature predominantly stems from the diminishment in dislocation density. And the reduction in dislocation density can be attributed to the attenuated interplay between dislocations and precipitates, as well as the alleviation of residual stresses and the genesis of reverse-transformed austenite through the tempering process.

## 4. Conclusions

This study scrutinizes the impact of tempering temperature on the mechanical characteristics, work hardening phenomena, and rotating bending fatigue of 40CrNi2MoE ultra-high-strength steel, and a succinct examination of the contributory role of dislocation density is presented. The following conclusions can be drawn:

- The present study was undertaken to scrutinize the impact of tempering temperature upon the mechanical attributes of 40CrNi2MoE steel. Notably, subsequent to tempering at 400 °C, the tensile strength of 40CrNi2MoE steel attains its zenith, registering at Rm = 1530 MPa. With the incremental elevation in the tempering temperature, both the strength and yield ratio manifest a discernible decrement, predominantly ascribed to the diminishment in dislocation density. Moreover, it is noteworthy that toughness evinces a correlation with the tempering temperature.
- A modified C–J analysis illuminates that, concomitant with the escalation in tempering temperature, the strain-hardening capacity experiences a gradual attenuation. Dislocations, together with precipitations, along with their interplay, constitute the chief determinants governing the strain-hardening demeanor of the examined steel.
- Under the purview of experimental conditions, the fatigue life of 40CrNi2MoE steel attains its apogee, nearing 320,000 cycles, following tempering at 400 °C. As the tempering temperature ascends, the fatigue life precipitously plummets, in tandem with the trend in the fluctuation of strength. This phenomenon predominantly hinges on the density of dislocations. Additionally, the three-parameter model presents a congruence with the S-N curve.
- The predominant origins of fatigue initiation in the context of rotational bending were inclusions, such as Al_2_O_3_, CaO, and MgO. Remarkably, the tempering temperature exerts no significant sway upon either the fount of initiation or the fracture morphology.

## Figures and Tables

**Table 1:** Composition of 40CrNi2MoE (mass fraction, %).

C	Si	Mn	Cr	Ni	Mo	Fe
0.41	0.25	0.78	0.85	1.86	0.26	Bal.

**Table 2:** Strain hardening rates and turning point strain of different stages.

Tempering Temperature/°C	n		
	I	II	III
400	21.07	39.86	2132
420	24.17	36.93	1445
440	23.47	30.51	580
460	24.03	41.00	1872

**Table 3:** Turning point strain of different stages.

Tempering Temperature/°C	$\varepsilon_{t}$/%	
	I→II	II→III
400	0.064	0.130
420	0.070	0.100
440	0.220	0.245
460	0.073	0.011

## Data Availability

All the data that support the findings of this study are available upon reasonable request.
